# Supplementary material for: Antigen recognition detains CD8+ T cells at the blood-brain barrier and contributes to its breakdown
Source: Nat Commun. 2023 May 30;14:3106. doi: 10.1038/s41467-023-38703-2 (PMC10229608; doi:10.1038/s41467-023-38703-2)
Supplement: Supplementary file 1 — Supplementary Information File [file 41467_2023_38703_MOESM1_ESM.pdf]

Antigen-recognition detains CD8$^+$ T cells at the blood-brain barrier and contributes to its breakdown

Sidar Aydin[1&], Javier Pareja[1&], Vivianne M. Schallenberg[1], Armelle Klopstein[1], Thomas Gruber[2], Nicolas Page[3], Elisa Bouillet[1], Nicolas Blanchard[4], Roland Liblau[4], Jakob Körbelin[5], Markus Schwaninger[6], Aaron J. Johnson[7], Mirjam Schenk[2], Urban Deutsch[1], Doron Merkler[3], Britta Engelhardt[1]*

[1]Theodor Kocher Institute, University of Bern, Bern, Switzerland

[2]Institute of Pathology, Experimental Pathology, University of Bern, Bern, Switzerland

[3]Department of Pathology and Immunology, Division of Clinical Pathology, University and University Hospitals of Geneva, Geneva, Switzerland

[4]Toulouse Institute for infectious and inflammatory diseases, University of Toulouse, CNRS, INSERM, UPS, Toulouse, France

[5]Department of Oncology, Hematology and Bone Marrow Transplantation, University Medical Center Hamburg-Eppendorf, Hamburg, Germany

[6]Institute for Experimental and Clinical Pharmacology and Toxicology, Center of Brain, Behavior and Metabolism, University of Lübeck, Lübeck, Germany

[7]Mayo Clinic Graduate School of Biomedical Sciences, College of Medicine, Mayo Clinic, Rochester, MN, USA

[&] Contributed equally

***Corresponding author:**
Britta Engelhardt
E-Mail: britta.engelhardt@tki.unibe.ch
ORCID:orcid.org/0000-0003-3059-9846

Supplementary Information

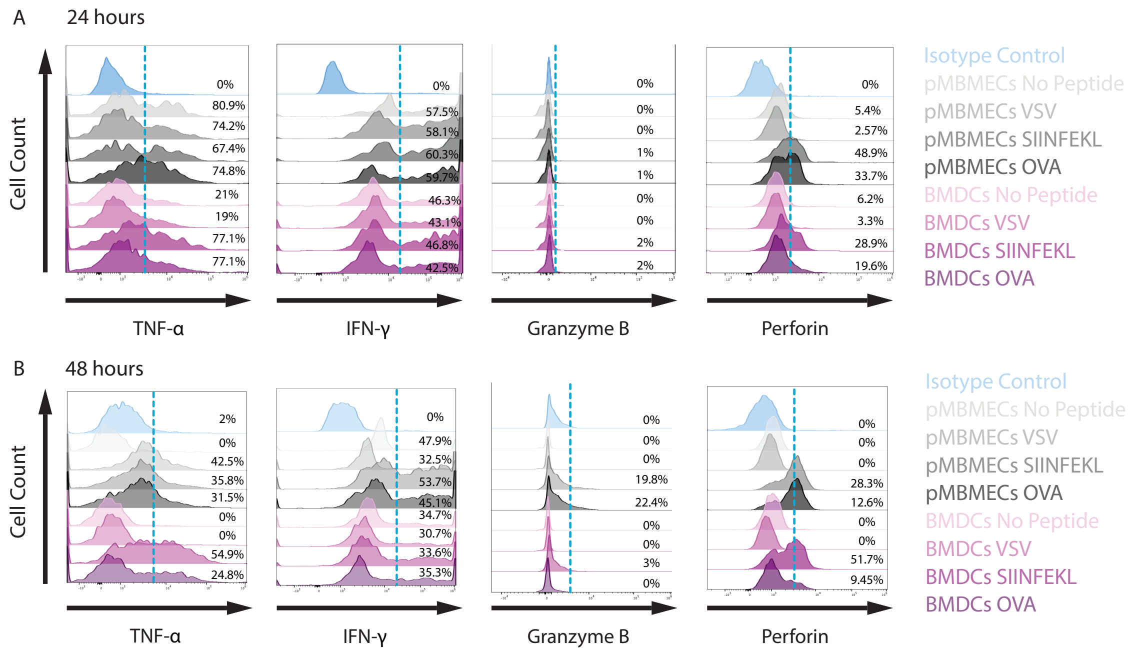

Supplementary Figure 1. Phenotype of OT-I cells upon-co-culture with WT pMBMECs
(A-B) Flow cytometry analysis of naïve OT-I cells after 24 **(A)** and 48 hours **(B)** hours of co-culture with TNF-α/IFN-γ stimulated WT pMBMECs in the absence of antigen, or in the presence of VSV- or SIINFEKL peptide or full-length ovalbumin (OVA) protein. Histograms show cell immunostaining of $CD3^{+}CD8^{+}$ OT-I cells for the cytotoxicity related molecules granzyme B, perforin and the cytokines TNF-α and IFN-γ. Isotype control for each marker in each condition is shown at the top of each plot. Percentage of events above the dashed blue threshold is indicated. The data is representative of 1 individual experiment. Gating strategy for OT-I cells is shown in Supplementary Figure 2. Source data are provided as a Source Data file.

OT-I T cells after co-incubation with WT pMBMECs (72h)

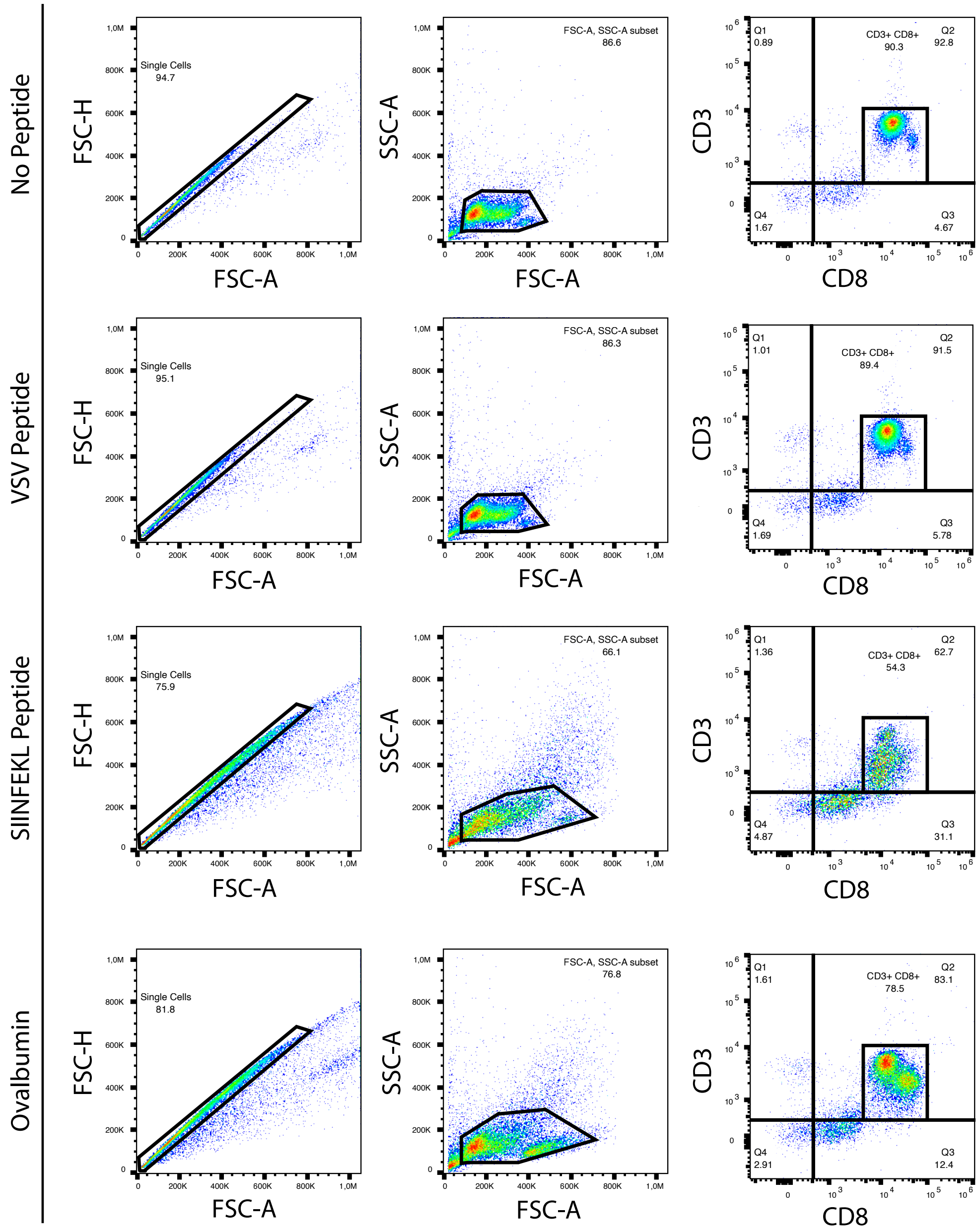

Supplementary Figure 2. Gating strategy for OT-I cells upon co-culture with WT pMBMECs

Flow cytometry analysis of naïve OT-I cells after 24 hours of co-culture with TNF-α/IFN-γ stimulated WT pMBMECs in the absence of antigen, or in the presence of VSV- or SIINFEKL peptide or full-length ovalbumin (OVA) protein. Single cells are selected first by using forward scatter height versus forward scatter area (FSC-H::FSC-A). $CD3^+CD8^+$ cells were selected from the population of interest based on the side scatter area versus forward scatter area (SSC-A::FSC-A). OT-I $CD8^+$ T cells that are co-cultured with unpulsed or VSV-pulsed pMBMECs did not show an increase in their size and granulation, whereas SIINFEKL pulsed- or OVA-loaded pMBMECs induced a major increase in the size and granulation of the naïve OT-I $CD8^+$ T cells, indicating their activation. Moreover, activated OT-I $CD8^+$ T cells exhibited decreased $CD3^+$ cell surface expression.

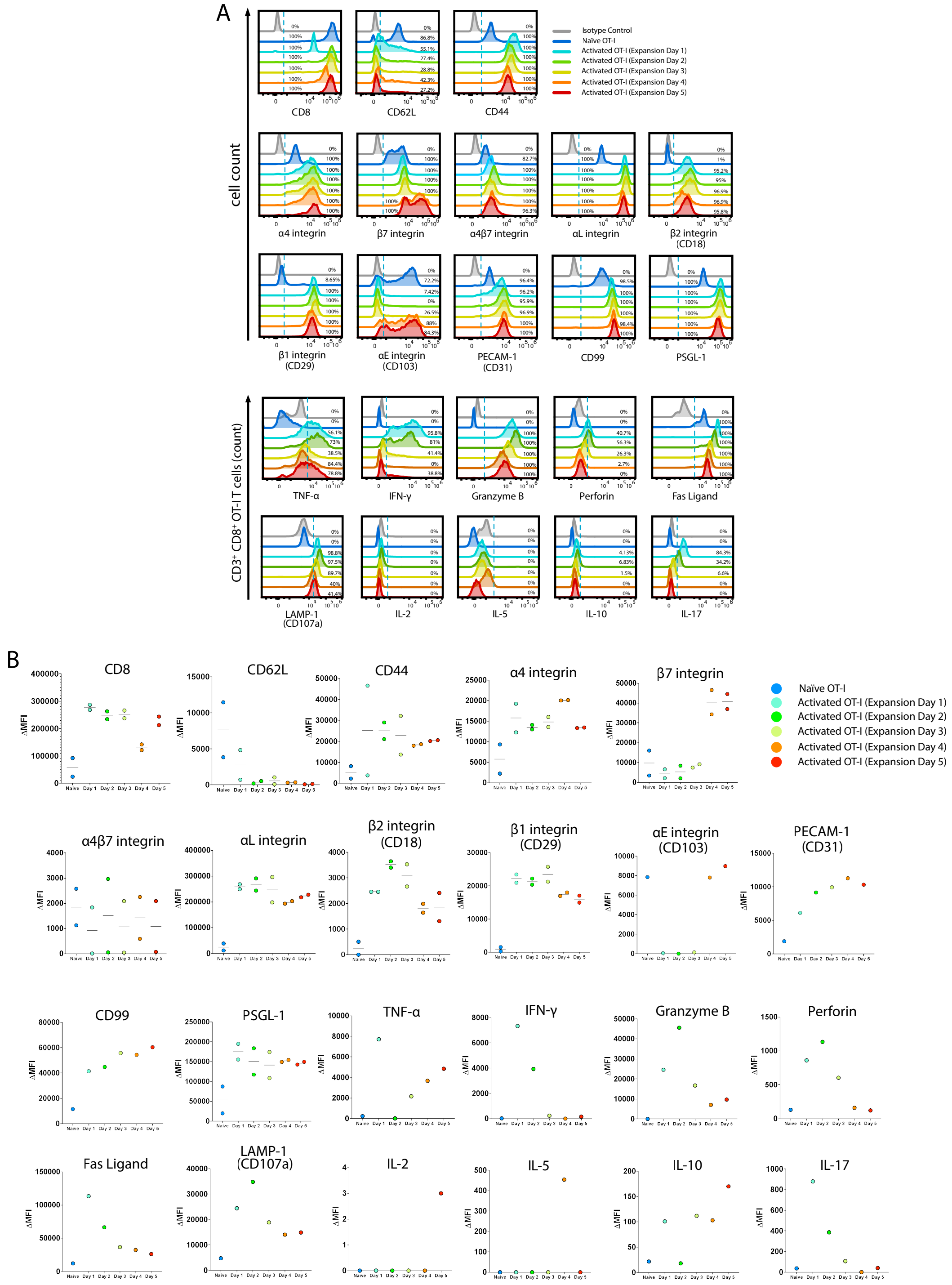
A
cell count
CD3+ CD8+ OT-I T cells (count)
Isotype Control
Naïve OT-I
Activated OT-I (Expansion Day 1)
Activated OT-I (Expansion Day 2)
Activated OT-I (Expansion Day 3)
Activated OT-I (Expansion Day 4)
Activated OT-I (Expansion Day 5)
CD8
CD62L
CD44
α4 integrin
β7 integrin
α4β7 integrin
αL integrin
β2 integrin (CD18)
β1 integrin (CD29)
αE integrin (CD103)
PECAM-1 (CD31)
CD99
PSGL-1
TNF-α
IFN-γ
Granzyme B
Perforin
Fas Ligand
LAMP-1 (CD107a)
IL-2
IL-5
IL-10
IL-17
B
ΔMFI
Naïve OT-I
Activated OT-I (Expansion Day 1)
Activated OT-I (Expansion Day 2)
Activated OT-I (Expansion Day 3)
Activated OT-I (Expansion Day 4)
Activated OT-I (Expansion Day 5)
Naive
Day 1
Day 2
Day 3
Day 4
Day 5

Supplementary Figure 3. Phenotype of *in vitro* activated OT-I cells

Phenotype of *in vitro* activated and expanded OT-I cells by flow cytometry at different stages of their expansion. Naïve OT-I cells were used to compare the changes following the *in vitro* activation. **(A)** Single color flow cytometry staining of OT-I cells for activation and cell surface expression of T cell trafficking related markers: CD8, CD44, CD62L, α4-, ß7-, α4ß7-, αL-, ß2-, αE-, ß1-integrins, PECAM-1, CD99, PSGL-1, as well as for cytotoxicity related molecules: granzyme B, perforin, Fas ligand, LAMP-1 and cytokines TNF-α, IFN-γ, IL-2, IL-5, IL-10, IL-17. Percentage of events above the dashed blue threshold is indicated. At the expansion day 1, OT-I cells are not fully activated based on the loss of cell surface staining for CD62L, while at the expansion day 4 and 5, OT-I T cells show the upregulation of αE-integrin, indicating a tissue memory cell phenotype. The data is representative of 1 individual experiment for αE-integrin, CD99, granzyme B, perforin, Fas ligand, LAMP-1, TNF-α, IFN-γ, IL-2, IL-5, IL-10, IL-17 and of 2 individual experiments for CD8, CD44, CD62L, α4-, ß7-, α4ß7-, αL-, ß2-, ß1-integrins and PSGL-1. **(B)** Median fluorescence intensity normalized to the isotype control in (A). Source data from B are provided as a Source Data file.

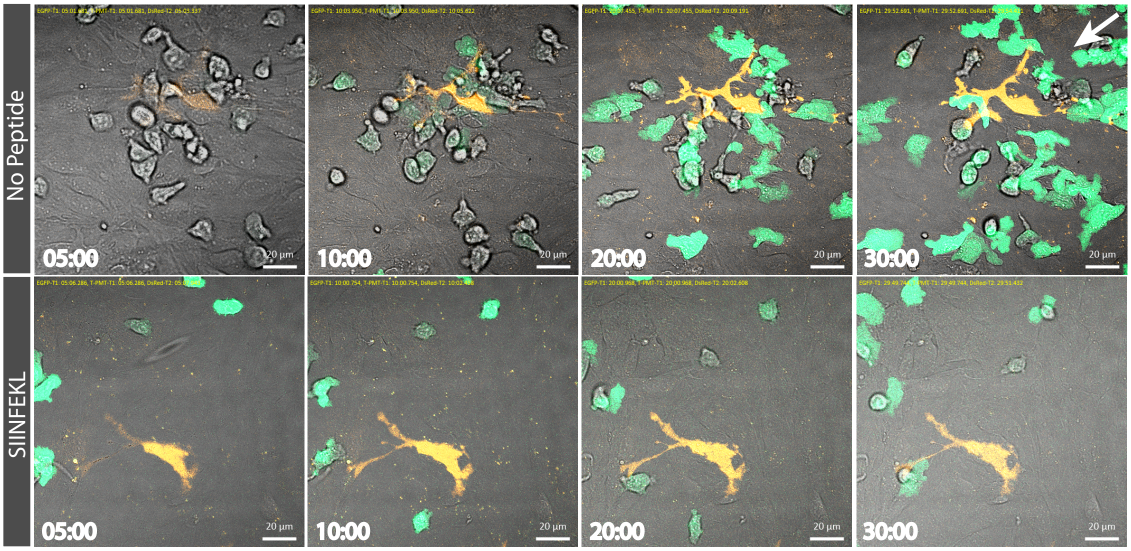

Supplementary Figure 4. OT-I cells do not interact with pericytes in pMBMEC monolayers in an MHC class I restricted and Ag-dependent manner under physiological flow

Interaction of CMFDA-labeled activated OT-I T cells with TNF-α/IFN-γ stimulated pMBMECs from NG2-DsRed C57BL/6J mice pulsed or not with SIINFEKL peptide at different timepoints under physiological flow is shown. $1x10^6$ OT-I cells were superfused over the pMBMECs. Images are recorded for 30 minutes with LSM 800 confocal microscope (Carl Zeiss, Oberkochen, Germany). White arrow indicates the direction of the flow. OT-I T cells (green) do not specifically interact with the pericyte (orange) in the SIINFEKL-pulsed pMBMEC monolayer. Data are representative of 3 independent experiments. Scale bar = 20 µm.

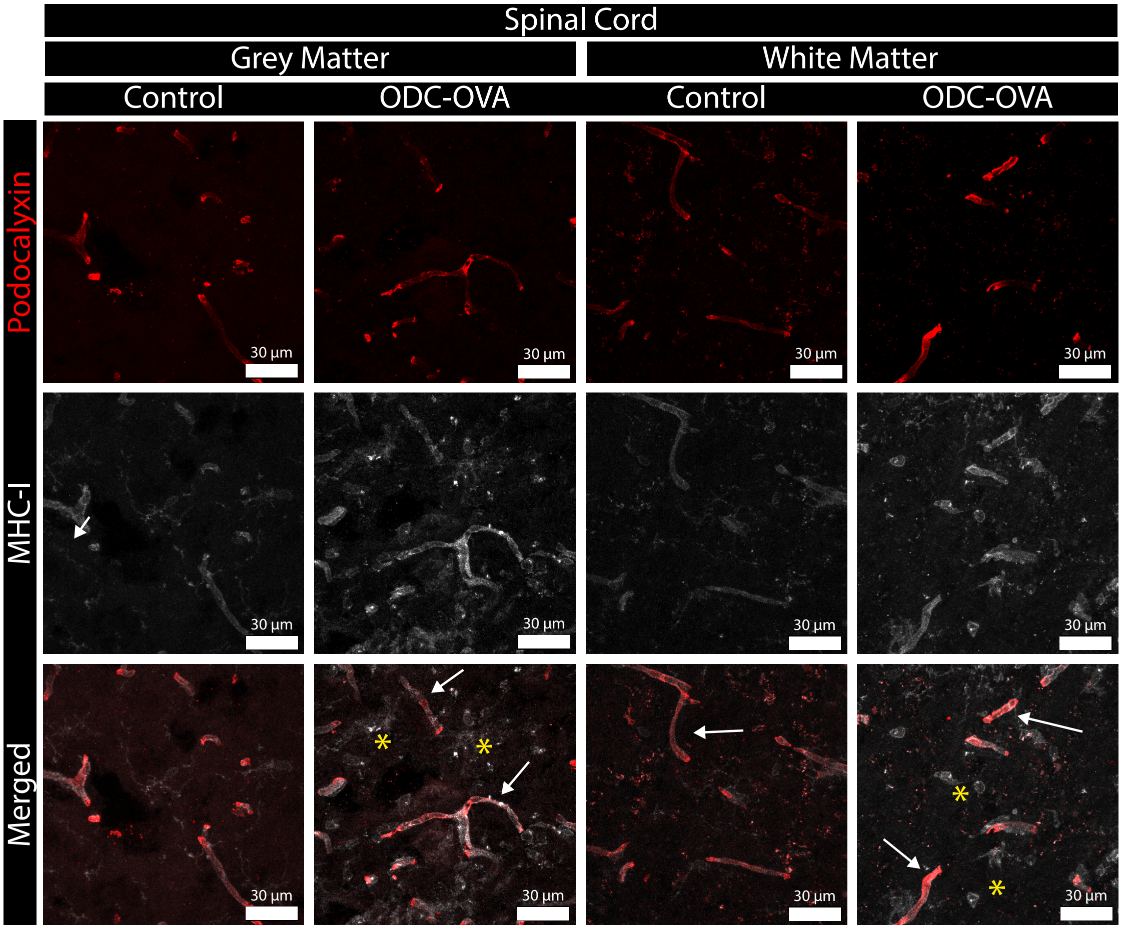

Supplementary Figure 5. MHC-I colocalization with CNS vasculature in the spinal cord of ODC-OVA and WT C57BL/6J mice.

Immunofluorescence staining of WT C57BL/6J and ODC-OVA mice spinal cords on day 7 after LCMV-OVA infection for MHC class I (grey), and endothelial cells (podocalyxin, red). Stainings in grey matter are displayed in the left panel and in the white matter on the right panel. White arrows show CNS vessels with a signal from both MHC class I and podocalyxin. Purple asterisks point to other CNS resident cells staining positive for MHC class I molecules on their surface. Data are representative of 5 different stainings from 3 mice from 3 independent experiments. Scale bar = 30 µm

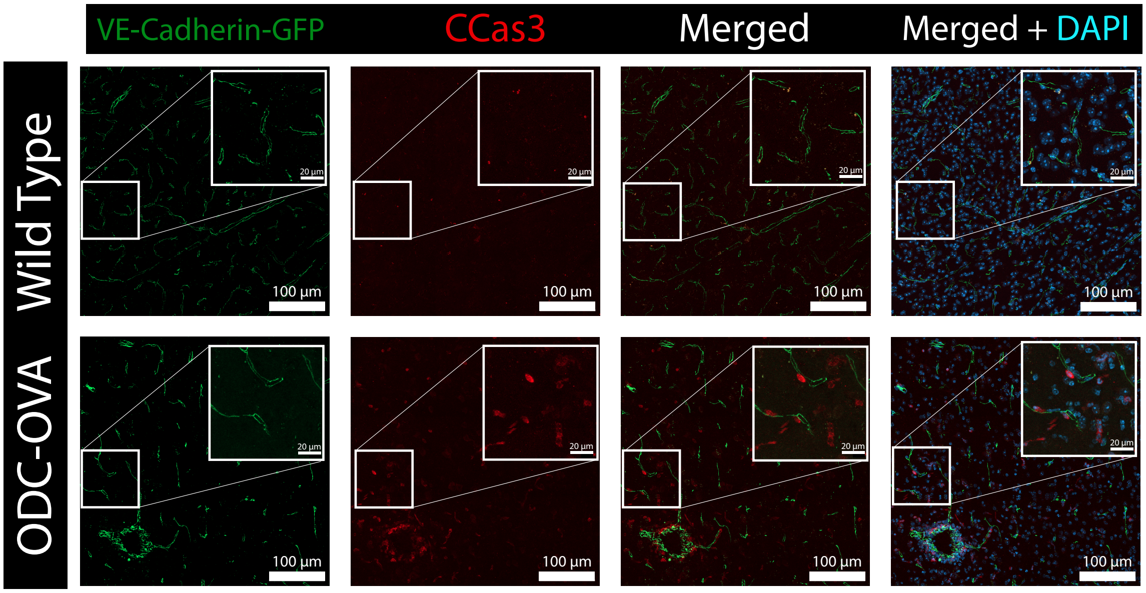

Supplementary Figure 6. CD8⁺ T cell driven neuroinflammation induces apoptotic events associated to the CNS vasculature

Brains from VE-Cadherin-GFP (Wild Type) or VE-Cadherin-GFP ODC-OVA mice were harvested on day 7 after LCMV-OVA infection. 20 µm cryosections were stained for Cleaved Caspase 3 (CCas3) as a marker for apoptosis (red) and nuclei (DAPI, blue). Areas within white boxes are shown magnified on the top right allowing to depict the presence of CCas3 positive perivascular cells in VE-Cadherin-GFP ODC-OVA but not in VE-Cadherin-GFP control mice. Data is representative of 3 independent experiments. Scale bar = 100 µm.

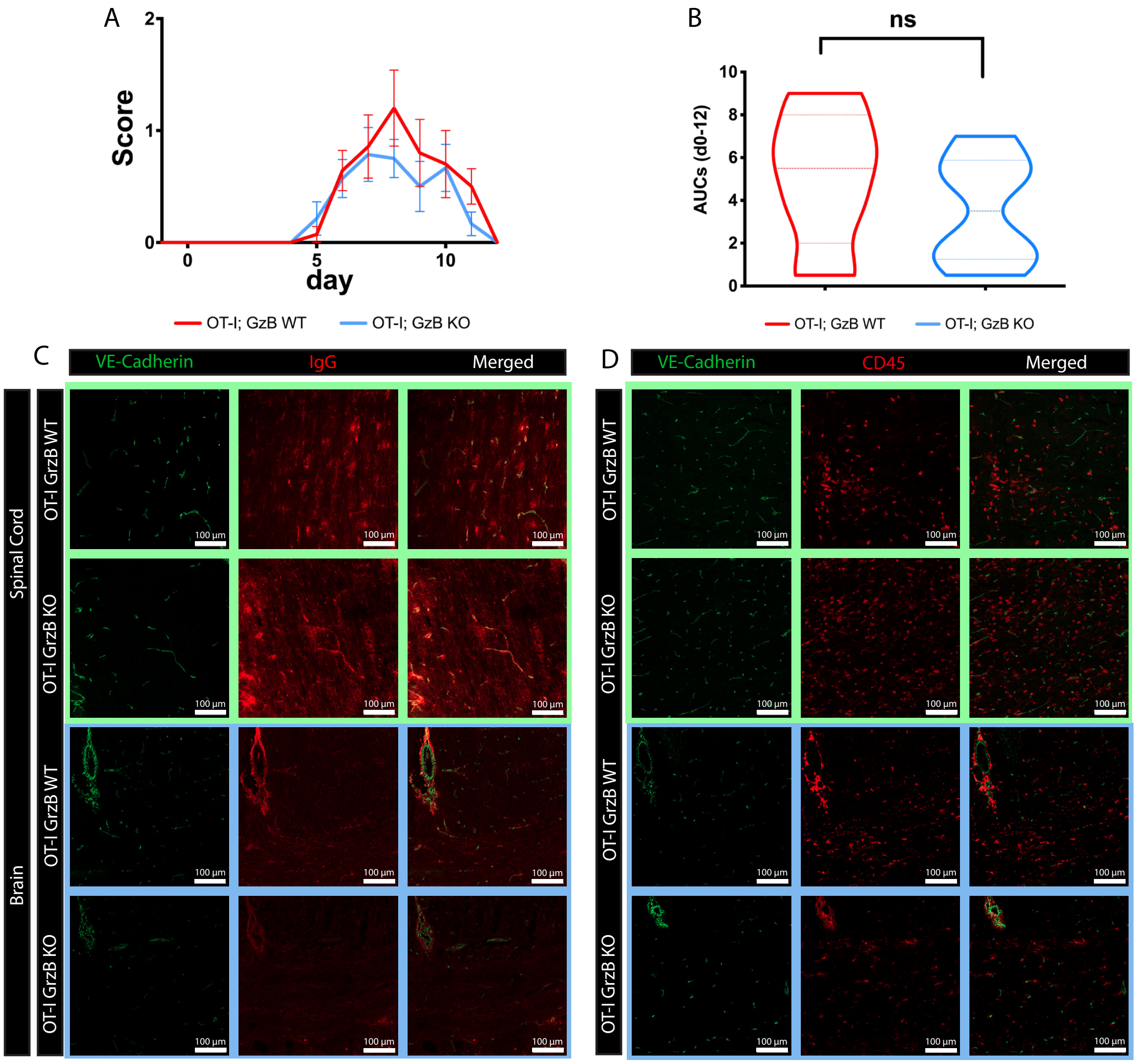

Supplementary Figure 7. Granzyme B is dispensable for OT-I induced autoimmune neuroinflammation ODC-OVA mice

OT-I cell driven neuroinflammation was induced in VE-Cadherin-GFP ODC-OVA mice by transferring naïve OT-I or naïve OT-I $GrB^{-/-}$ cells one day prior to LCMV-OVA infection. (**A**) Clinical disease course of VE-Cadherin-GFP ODC-OVA mice after injection of OT-I GrB WT (red; n=7)) or OT-I GrB KO cells (blue; n= 7) shown as mean ± SD. Data were analyzed using two-sided unpaired parametric T-test. **(B)** Overall clinical severity of VE-Cadherin-GFP ODC-OVA mice after injection of OT-I GrB WT (red, n=7) or OT-I GrB KO cells (blue; n=7) shown as area under the curve. Data were analyzed using two-sided unpaired parametric T-test. (**C**, **D**) Brains (blue box) and spinal cords (green box) from ODC-OVA mice in which autoimmune neuroinflammation was induced with either OT-I WT or OT-I GrB KO followed by LCMV-OVA infection were harvested on day 7 after infection. Immunofluorescence staining for IgG (**C**) or CD45 (**D**) (red) was performed on 20 μm thick cryosections. VE-cadherin-GFP visualizes vascular adherens junctions. Data are representative of 3 independent mice. Scale bar = 100 μm. Source data from A and B are provided as a Source Data file.

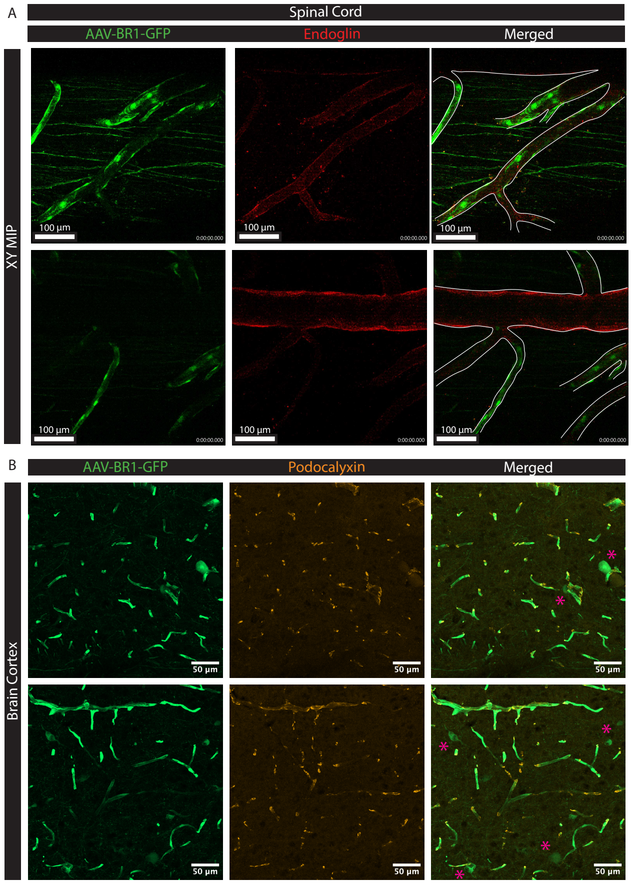

Supplementary Figure 8. The AAV-BR1 vector shows a high tropism CNS microvascular endothelial cells. (**A**) Representative images of 2P-IVM imaging of the cervical spinal cord vessels (red) of an ODC-OVA TAP1$^{fl/fl}$ mouse on day 7 after LCMV-OVA injection. The mice were infected with AAV-BR1-GFP two weeks prior to the induction of autoimmune neuroinflammation. AAV-BR1-GFP infected cells are shown in green. The pictures are depicted as maximum intensity projection (MIP) of 100 μm thick Z-stacks. (**B**) Immunofluorescence staining for podocalyxin (orange) on 20 μm thick cryosection from brains isolated from an AAV-BR1-GFP infected ODC-OVA TAP1$^{fl/fl}$ mouse on day 7 after LCMV-OVA injection. AAV-BR1-GFP infected cells are shown in green. Magenta asterisks point to non-endothelial CNS resident cells infected with the AAV-BR1-GFP virus. Data are representative for 3 individual mice.

Supplementary Table 1. Sequence of primers used for the qPCR analysis.

Official Gene Name	Entrez Gene ID	Accession Number	Primer Sequences		Supplier
CD80 (mouse)	12519	NM_009855	Quantitech primer assay for mouse CD80, QT00129787		Qiagen
CD86 (mouse)	12524	NM_019388	Quantitech primer assay for mouse CD86, QT01055250		Qiagen
H2-K1 (mouse)	14972	NM_001001892	Quantitech primer assay for mouse H2K1, QT01743700		Qiagen
CD274 (PD-L1) (mouse)	60533	NM_021893	Quantitech primer assay for mouse PD-L1, QT01743700		Qiagen
**β-actin (mouse)** (endogenous control)	11461	NM_007393	Sense	5'- CGTGGGCCGCCCTAGGCACCA -3’	Eurogentec
			Anti sense	5'- TTGGCTTAGGGTTCAGGGGGG -3’	

Supplementary Table 2. List of antibodies used in this study.

Flow Cytometry Antibodies				
**Primary Antibodies**	**Company**	**CAT Nr.**	**Working Conc.**	**Clone**
Rat monoclonal anti - Mouse CD25 - FITC	BioLegend	102006	5 μg/mL	PC61
Armenian Hamster monoclonal anti- Mouse CD3 - APC	BioLegend	100312	5 μg/mL	145-2C11
Armenian Hamster monoclonal anti - Mouse CD3 - BV605	BioLegend	100351	8 μg/mL	145-2C11
Rat monoclonal anti - Mouse CD44 - eFluor450	Thermo Fisher Scientific	48-0441-80	5 μg/mL	IM7
Rat monoclonal anti - Mouse CD62L - PE	BioLegend	104408	0.2 μg/mL	MEL-14
Armenian Hamster monoclonal anti - Mouse CD69 - BV711	BioLegend	104537	4 μg/mL	H1-2F3
Rat monoclonal anti - Mouse CD8 - PE-Cy7	BioLegend	100722	6 μg/mL	53-6.7
Rat monoclonal - Mouse CD8 - PerCP	BioLegend	100732	2 μg/mL	53-6.7
Mouse monoclonal anti - Mouse FasL - PE	BioLegend	106805	10 μg/mL	Kay-10
Mouse monoclonal anti - Mouse Granzyme B - FITC	BioLegend	515403	10 μg/mL	GB11
Rat monoclonal anti - Mouse IFN-gamma - BV711	BioLegend	505836	10 μg/mL	XMG1.2
Rat monoclonal anti - Mouse LAMP-1 (CD107a) - PerCP-eFluor710	Thermo Fisher Scientific	46-1071-80	10 μg/mL	eBio1D4B (1D4B)
Rat monoclonal anti - Mouse Perforin - APC	Thermo Fisher Scientific	17-9392-80	10 μg/mL	eBioOMAK -D
Rat monoclonal - Mouse TNF-alpha - BV510	BD Biosciences	563386	5 μg/mL	MP6-XT22
Monoclonal Armenian Hamster IgG - BV605	BioLegend	400944	8 μg/mL	HTK888
Monoclonal Rat IgG2a - PE-Cy7	Thermo Fisher Scientific	25-4321-81	6 μg/mL	eBR2a
Monoclonal Rat IgG1 - BV510	BD Biosciences	563039	5 μg/mL	R3-34
Monoclonal Rat IgG1 - BV711	BioLegend	400441	4 μg/mL	RTK2071
Monoclonal Mouse IgG1 - FITC	BD Biosciences	554679	10μg/mL	MOPC-21
Monoclonal Armenian Hamster IgG - APC	BioLegend	400911	10μg/mL	HTK888
Monoclonal Rat IgG2b - eFluor450	Thermo Fisher Scientific	48-4031-80	5 μg/mL	eB149/10H 5
Monoclonal Rat IgG2a - PE	BioLegend	400508	0.2 μg/mL	RTK2758
Monoclonal Rat IgG2a - PerCP	BioLegend	400530	2 μg/mL	RTK2758
Monoclonal Mouse IgG2b - PE	BioLegend	400314	10 μg/mL	MPC-11

Monoclonal Armenian Hamster IgG - BV711	BioLegend	400963	10µg/mL	HTK888
Monoclonal Rat IgG2a - PerCP - eFluor710	Thermo Fisher Scientific	46-4321-80	10 µg/mL	eBR2a
Monoclonal Rat IgG2a - APC	BioLegend	400512	5 µg/mL	RTK2758

**Immunofluorescence Staining Antibodies**				
**Primary Antibodies**	**Company**	**CAT Nr.**	**Working Conc.**	
Rat monoclonal anti - Mouse CD8	BioLegend	100702	10 µg/mL	53-6.7
Armenian Hamster monoclonal anti - Mouse CD80	BioLegend	104702	10 µg/mL	16-10A1
Rat monoclonal anti - Mouse CD86	BioLegend	105002	10 µg/mL	GL-1
Rat monoclonal anti - Mouse MHC class I	BMA Biomedicals	T-2105	20 µg/mL	ER-HR52
Rat monoclonal anti - Mouse PD-L1	BioLegend	124302	2.0 µg/mL	10F.9G2
Goat polyclonal anti - Mouse Podocalyxin	R&D Systems	AF1556	2.0 µg/mL	Polyclonal
Rabbit polyclonal anti - Mouse ZO-1	Thermo Fisher Scientific	61-7300	1.25 µg/mL	Polyclonal
Rat monoclonal anti - Rat IgG2a	BioLegend	400501	10 µg/mL	RTK2758
Monoclonal Armenian Hamster IgG	BioLegend	400901	10 µg/mL	HTK888
Monoclonal Rat IgG2b	BioLegend	400601	2.0 µg/mL	RTK4530
Monoclonal mouse IgG2a	abcam	ab18415	2.0 µg/mL	MG2a-53
Purified rat anti- mouse CD40 (3/23)	BD Pharmingen	553788	10 µg/mL	3/23
Policlonal Rabbit anti-laminin	DAKO	Z0097	10 µg/mL	Polyclonal
Rat anti-mouse CD45	In house	-	2 µg/mL	M1/9.3.4.HL.2
Rat anti- mouse VCAM-1	In house	-	20 µg/mL	2A11.12
Rat anit- mouse ICAM-1	In house	-	6 µg/mL	25ZC7
Rabbit anti-mouse cleaved caspase 3 (Asp175)	Cell Signaling	9661S	10 µg/mL	Polyclonal
Polyclonal Rabbit IgG	R&D Systems	AB-105-C	1.25 µg/mL	Polyclonal
**Secondary Antibodies**	**Company**	**CAT Nr.**	**Working Conc.**	
Donkey polyclonal anti - Rabbit IgG - AF488	Thermo Fisher Scientific	A-21206	10 µg/mL	Polyclonal
Goat polyclonal - anti - Rat IgG - Cy3	Jackson Immuno Research	112-165-143	2.5 µg/mL	Polyclonal
Goat polyclonal Biotin - anti - Hamster	BioLegend	405501	10 µg/mL	Polyclonal

Goat polyclonal - anti - Rabbit IgG - AF647	Thermo Fisher Scientific	A-21244	3 µg/mL	Polyclonal
Donkey polyclonal - anti - Goat IgG - Cy3	Jackson Immuno Research	705-165-147	5 µg/mL	Polyclonal
Donkey polyclonal - anti-Rat IgG - AF488	Thermo Fisher Scientific	A-21208	5 µg/mL	Polyclonal
Donkey anti-rat Cy5	Jackson Immuno Research	712-175-153	5 µg/mL	Polyclonal
Rabbit anti-Hamster Rhodamin	Pierce	31652	7.5 µg/mL	Polyclonal
donkey anti-goat AF647	Jackson Immuno Research	705-605-003	7.5 µg/mL	Polyclonal
Streptavidin Cy3	BioLegend	405215	10 µg/mL	-
