# Supplementary material for: Antigen recognition detains CD8+ T cells at the blood-brain barrier and contributes to its breakdown
Source: Nat Commun. 2023 May 30;14:3106. doi: 10.1038/s41467-023-38703-2 (PMC10229608; doi:10.1038/s41467-023-38703-2)
Supplement: Supplementary file 3 — Reporting Summary [file 41467_2023_38703_MOESM3_ESM.pdf]

nature portfolio

Corresponding author(s): Britta Engelhardt

Last updated by author(s): Apr 19, 2023

Reporting Summary

Nature Portfolio wishes to improve the reproducibility of the work that we publish. This form provides structure for consistency and transparency in reporting. For further information on Nature Portfolio policies, see our Editorial Policies and the Editorial Policy Checklist.

Statistics

For all statistical analyses, confirm that the following items are present in the figure legend, table legend, main text, or Methods section.

n/a	Confirmed	
☐	☒	The exact sample size (*n*) for each experimental group/condition, given as a discrete number and unit of measurement
☐	☒	A statement on whether measurements were taken from distinct samples or whether the same sample was measured repeatedly
☐	☒	The statistical test(s) used AND whether they are one- or two-sided<br>*Only common tests should be described solely by name; describe more complex techniques in the Methods section.*
☒	☐	A description of all covariates tested
☐	☒	A description of any assumptions or corrections, such as tests of normality and adjustment for multiple comparisons
☐	☒	A full description of the statistical parameters including central tendency (e.g. means) or other basic estimates (e.g. regression coefficient) AND variation (e.g. standard deviation) or associated estimates of uncertainty (e.g. confidence intervals)
☐	☒	For null hypothesis testing, the test statistic (e.g. *F*, *t*, *r*) with confidence intervals, effect sizes, degrees of freedom and *P* value noted<br>*Give P values as exact values whenever suitable.*
☒	☐	For Bayesian analysis, information on the choice of priors and Markov chain Monte Carlo settings
☒	☐	For hierarchical and complex designs, identification of the appropriate level for tests and full reporting of outcomes
☒	☐	Estimates of effect sizes (e.g. Cohen's *d*, Pearson's *r*), indicating how they were calculated

Our web collection on statistics for biologists contains articles on many of the points above.

Software and code

Policy information about availability of computer code

Data collection	Confocal image acquisition was performed with the Zeiss LSM800 microscope, controlled by the ZEN 2.6.76 software.<br>IF staining images were also acquired using a Nikon Eclipse E600 microscope connected to a Nikon Digital Camera DXM1200F with the Nikon NIS-Elements BR3.10 software.<br>Flow cytometry acquisition was performed with the Attune NxT from life technologies, controlled by the Attune NxT Software v3.1.2<br>Two photon image acquisition was performed with the Spectra Physics InSight X3 laser, controlled by the Imspector 5.1.333<br>Distorsion correction of the images during two-photon microscopy imaging was performed by using Vivo Follow 2.0 (Vladymyrov, M., Haghayegh Jahromi, N., Kaba, E., Engelhardt, B. & Ariga, A. VivoFollow 2: Distorsion-Free Multiphoton Intravital Imaging. Frontiers in Physics 7 (2020).<br>In vitro life cell imaging was performed with the inverted microscope, AxioObserver from Carl Zeiss.<br>Absorbance for BrdU incorporation assay was measured with Infinite 200 Tecan reader, controlled by the Infinite Pro 200 software.
Data analysis	Image analysis was performed using Image J/Fiji (version 2.3)<br>Intravital microscopy images were analysed with Imaris x64 9.1.2. Intravital microscopy cell tracks were obtained using the semiautomated spot function from Imaris x64 9.8<br>Cell tracks from in vitro videos were analysed using the Chemotaxis and Migration Tool V2.0 written in 2008-2010 by Gerhard Trapp.<br>Flow cytometry data was analyzed with FlowJo 10.7.1<br>Statistical analysis and data representation was performed with GraphPad Prism v 9.2.<br>Figures were made using Adobe Illustrator 25.4.1<br>Movies were processed with Adobe After Effects 18.4.1

For manuscripts utilizing custom algorithms or software that are central to the research but not yet described in published literature, software must be made available to editors and reviewers. We strongly encourage code deposition in a community repository (e.g. GitHub). See the Nature Portfolio guidelines for submitting code & software for further information.

Data

Policy information about availability of data

All manuscripts must include a data availability statement. This statement should provide the following information, where applicable:

- Accession codes, unique identifiers, or web links for publicly available datasets
- A description of any restrictions on data availability
- For clinical datasets or third party data, please ensure that the statement adheres to our policy

All data is made available as a Source Data File.

Research involving human participants, their data, or biological material

Policy information about studies with human participants or human data. See also policy information about sex, gender (identity/presentation), and sexual orientation and race, ethnicity and racism.

Reporting on sex and gender	n/a
Reporting on race, ethnicity, or other socially relevant groupings	n/a
Population characteristics	n/a
Recruitment	n/a
Ethics oversight	n/a

Note that full information on the approval of the study protocol must also be provided in the manuscript.

Field-specific reporting

Please select the one below that is the best fit for your research. If you are not sure, read the appropriate sections before making your selection.

☒ Life sciences ☐ Behavioural & social sciences ☐ Ecological, evolutionary & environmental sciences

For a reference copy of the document with all sections, see nature.com/documents/nr-reporting-summary-flat.pdf

Life sciences study design

All studies must disclose on these points even when the disclosure is negative.

Sample size	For the in vitro experiments a total of three technical replicates was performed within each assay for each value and three independent biological experiments per condition were performed. For the in vitro flow assays, a minimum of three biological replicates per experiments were performed until a minimum of 30 cells per condition could be analyzed. This sample size reached a high enough power to show statistical differences of the observed effects. A minimum of three individual mice per experimental condition were analyzed in the in vivo experiments again ensuring to yield a high enough number of events to show statistical differences between the different experimental groups. The numbers of mice imaged per condition and cells counted per FOV are within the ranges of comparable in vivo imaging studies in the field. Specific details are included in the figure legends.
Data exclusions	We did not exclude any data from consideration.
Replication	All the results reported in this study were successfully reproduced with the minimum of 3 biological replicates, this is independent experiments including different animals, different cell preparations etc.
Randomization	Randomization was applied when possible and applicable in this study. Treatment conditions were randomly assigned in the in vitro assay. In the in vivo experiments, mice within one experimental conditions were always randomized prior to the start of the experiment.
Blinding	Complete blinding of the experimenters was impossible as this would require duplication of staff with the respective expertise in two-photon imaging. Taking this into account, data analysis was regularly double checked for at least one experimental condition within one experiment by an additional experimenter not involved in this study.

Reporting for specific materials, systems and methods

We require information from authors about some types of materials, experimental systems and methods used in many studies. Here, indicate whether each material, system or method listed is relevant to your study. If you are not sure if a list item applies to your research, read the appropriate section before selecting a response.

Materials & experimental systems

n/a	Involved in the study
☐	☒ Antibodies
☒	☐ Eukaryotic cell lines
☒	☐ Palaeontology and archaeology
☐	☒ Animals and other organisms
☒	☐ Clinical data
☒	☐ Dual use research of concern
☒	☐ Plants

Methods

n/a	Involved in the study
☒	☐ ChIP-seq
☐	☒ Flow cytometry
☒	☐ MRI-based neuroimaging

Antibodies

Antibodies used

Detailed information about the antibodies used in this study are provided in Supplementary Table 1. The final concentration and dilution factors of IgG controls are stated in the table as variable because IgG controls are used in the same final concentration as the antibodies that they are controlling. Hence, if the same IgG control antibody is used for different primary antibodies, their final concentration changes accordingly.

Validation

All antibodies used are commercially available and have been validated by their manufacturers: Biolegend, Thermo Fischer Scientific and BD Biosciences. Data regarding their validation can be found in their webpages using the catalog number of each antibody provided in the Supplementary Table I.

Animals and other research organisms

Policy information about studies involving animals; ARRIVE guidelines recommended for reporting animal research, and Sex and Gender in Research

Laboratory animals

Male and female mice from C57BL/6J strain between 8-12 weeks of age were used in this study. Species, strains, sex and age of all laboratory animals used in this study are also adequately reported in the section describing the animals used in the Methods section. All mice were housed in individually ventilated cages under specific pathogen free conditions in a temperature-controlled room (22° C) with a 13:11 hour light cycle. They had ad libitum access to water and chow.

Wild animals

No wild animal was used in this study

Reporting on sex

Both male and female mice were used for the pMBMEC isolations and preparations.
For the in vivo experiments, only female mice were used due to the higher reproducibility of the neuroinflammation model in female mice as well as to avoid confounding effects from additional immune reactions after the T cell transfer from different donors from different sex.

Field-collected samples

No field-collected sample was used in this study

Ethics oversight

Animal procedures were approved by the Veterinary Office of the Canton Bern (permit no. BE31/17 and BE55/20) and are in line with institutional and standard protocols for the care and use of laboratory animals in Switzerland.

Note that full information on the approval of the study protocol must also be provided in the manuscript.

Flow Cytometry

Plots

Confirm that:

☒ The axis labels state the marker and fluorochrome used (e.g. CD4-FITC).

☒ The axis scales are clearly visible. Include numbers along axes only for bottom left plot of group (a 'group' is an analysis of identical markers).

☒ All plots are contour plots with outliers or pseudocolor plots.

☒ A numerical value for number of cells or percentage (with statistics) is provided.

Methodology

Sample preparation

Naïve CD8+ T cell isolation: Peripheral lymph nodes and spleens from Rag-1-/- OT-I, OT-I or tdTomato OT-I C57BL/6J mice were harvested and single cell suspensions were obtained by homogenization and filtration through a sterile 100 ?m nylon mesh. A second filtration was applied after erythrocyte lysis (0.83% NH_4Cl, Tris-HCl). OT-I cells were isolated with magnetic CD8+ T cell selection beads (EasySep, STEMCELL Technologies). The purity of the CD8+ T cells was assessed by flow cytometry and was >98.5% in each experiment.

In vitro activation of naïve CD8+ T cells: OT-I CD8+ T cells were isolated and activated from OT-I, tdTomato OT-I, Perforin-/-

OT-I or Granzyme B-/- OT-I mice exactly as described before (9, 59). Activated T cells were cultured in IL-2 containing media for 3 days post-activation.

In vivo activation of naïve CD8+ T cells: 2x105 naïve td-Tomato OT-I cells were intravenously (i.v.) injected into WT C57BL/6J mice 24 hours prior to intraperitoneal (i.p) infection with 105 plaque-forming unit (PFU), OVA expressing lymphocytic choriomeningitis virus (LCMV-OVA) (21). Spleens of recipient mice were collected 8 days after viral infection and CD8+ T cells were purified by magnetic bead selection (EasySep, STEMCELL Technologies). Fluorescence-activated cell sorting is used for the separation of tdTomato OT-I CD8+ cells from the CD8+ T cells of recipient WT mice.

Instrument	Flow cytometry acquisition was performed with Attune NxT from life technologies, controlled by the Attune NxT Software v3.1.2
Software	Flow cytometry data was analyzed with FlowJo 10.7.1
Cell population abundance	The abundance of the CD8+ T cell population analyzed after the isolation from the spleen and lymph nodes used for the experiments in this study ranged from 50-70%.
Gating strategy	Single cells were selected first by using forward scatter height versus forward scatter area (FSC-H::FSC-A). CD3+CD8+ cells were selected from the population of interest based on the side scatter area versus forward scatter area (SSC-A::FSC-A). See supplementary figure 2.

☒ Tick this box to confirm that a figure exemplifying the gating strategy is provided in the Supplementary Information.
