# Supplementary material for: Antigen recognition detains CD8+ T cells at the blood-brain barrier and contributes to its breakdown
Source: Nat Commun. 2023 May 30;14:3106. doi: 10.1038/s41467-023-38703-2 (PMC10229608; doi:10.1038/s41467-023-38703-2)
Supplement: Supplementary file 4 — Description of Additional Supplementary Files [file 41467_2023_38703_MOESM4_ESM.docx]

**Description of Additional Supplementary Files**

Supplementary Movie 1

Description: Priming of naïve OT-I cells by pMBMECs upon endothelial Ag-presentation. Time lapse imaging of TNF-α/IFN-γ stimulated, unpulsed or SIINFEKL-pulsed pMBMECs from VE-cadherin-GFP C57BL/6J mice with tdTomato expressing naïve OT-I CD8+ T cells under static conditions for 72 hours. Images are acquired by using a high content imaging system INCell Analyzer 2000 every 30 minutes. Cytokine stimulated-, peptide pulsed pMBMECs without T cells are used as an internal control. tdTomato expressing OT-I cells that are co-cultured with SIINFEKL-pulsed pMBMECs show an increase in size, indicating their activation and is followed by their proliferation. Activated OT-I cells fully disrupt the pMBMECs monolayer that is indicated by the loss of GFP signal at the junctions after their interactions. On the other hand, naïve OT-I cells that are co-cultured with unpulsed pMBMECs lose their cytoplasmic expression of tdTomato and die over 72 hours, while the pMBMECs remain intact. Cytokine stimulated-, SIINFEKL pulsed pMBMECs remain intact in the absence of CD8+ T cells.

Supplementary Movie 2

Description: Naïve OT-I cell interactions with pMBMECs under physiological flow Time lapse imaging of TNF-α/IFN-γ stimulated, VSV- or SIINFEKL-pulsed pMBMECs from WT or B2M-/- C57BL/6J mice with naïve OT-I cells under physiological flow for 30 minutes. Images are acquired with 10 seconds intervals. Different post-arrest behaviors are labeled with different circles (Probing: Green; Crawling: Orange; Detachment: Pink). Yellow arrow indicates the direction of the flow. The video shows that in the presence of endothelial Ag-presentation (SIINFEKL pulsed WT pMBMECs; lower left frame), the majority of the naïve OT-I cells stop and probe the endothelium upon recognition of the cognate antigen under physiological flow. Scale bar = 50 μm.

Supplementary Movie 3

Description: Effector OT-I cell interactions with pMBMECs under physiological flow Time lapse imaging of TNF-α/IFN-γ stimulated, VSV- or SIINFEKL-pulsed pMBMECs from WT or B2M-/- C57BL/6J mice with in vitro activated OT-I cells under physiological flow for 30 minutes. Images are acquired with 10 second intervals. Different post-arrest behaviors are labeled with different circles (Probing: Green; Crawling: Orange; Diapedesis: Blue). Yellow arrow indicates the direction of the flow. The video shows that in the presence of endothelial Ag-presentation (SIINFEKL pulsed WT pMBMECs; lower left frame) the majority of the effector OT-I cells stop and probe the endothelium upon recognition of the cognate antigen under physiological flow. White arrow heads show the pMBMEC monolayer disruption by the OT-I cells. Scale bar = 50 μm.

Supplementary Movie 4

Description: OT-I cell induced apoptosis of Ag-presenting WT pMBMECs under physiological flow Time lapse imaging of TNF-α/IFN-γ stimulated, SIINFEKL-pulsed pMBMECs from WT C57BL/6J mice with in vitro activated OT-I cells under physiological flow for 1 hour. Images are acquired with 10 second intervals. Yellow arrow indicates the direction of the flow. The video shows OT-I cell induced Agdependent pMBMEC disruption under physiological flow. Scale bar = 50 μm.

Supplementary Movie 5

Description: OT-I vs OT-I GrB-/- cell interactions with VECadherin-GFP pMBMECs under physiological flow Time lapse imaging of TNF-α/IFN-γ stimulated, unpulsed or SIINFEKL-pulsed pMBMECs from VE-Cadherin GFP C57BL/6J mice with in vitro activated effector OT-I or OT-I GrB-/- cells under physiological flow for 60 minutes. Yellow circle highlights the disruption of the junctions and the pMBMEC-monolayer. Scale bar = 20 μm.

Supplementary Movie 6

Description: Effector OT-I cell interactions with mixed Agpresentation competent and incompetent pMBMEC monolayer under physiological flow Time lapse imaging of OT-I cell interaction with TNF-α/IFN-γ stimulated, SIINFEKL-pulsed pMBMEC monolayers consisting of a mix of Ag-presentation competent Life-Act-GFP+ pMBMECs and Ag-presentation incompetent B2M-/- pMBMECs under physiological flow for 60 minutes is shown. Images are acquired with 10 second intervals. Yellow arrow indicates the direction of the flow. Scale bar = 100 μm.

Supplementary Movie 7

Description: OT-I CD8+ T cell probing on the BBB under neuroinflammation Time lapse imaging of two-photon intravital microscopy of in vivo activated OT-I cells (red) and Cell Tracker Green CMFDA labeled in vitro activated effector OT-I cells (green) adhered in inflamed cervical spinal cord microvessels (white) of WT and ODCOVA C57BL/6J mice over 30 minutes on day 7 after LCMV-OVA infection. CMFDA labelled effector OT-I cells were injected via a carotid artery catheter immediately prior to the imaging. For 2PM-IVM the blood vessels were in vivo stained with Alexa Fluor 633- conjugated rat-anti mouse endoglin antibody (20 µg/mouse). The video is depicted as maximum intensity projection of 100 μm thick Z-stack. The blue circles indicate in vitro activated CD8 T cells arrested on the BBB. Scale bar = 50 μm.
